# A Feasibility Study on Monitoring Earthquake-Caused Furniture Vibrations Using Radiofrequency Identification Sensor Tags

**DOI:** 10.3390/s23063279

**Published:** 2023-03-20

**Authors:** Zequn Song, Budi Rahmadya, Ran Sun, Shigeki Takeda

**Affiliations:** 1 Graduate School of Science and Engineering, Ibaraki University, Hitachi 316-8511, Japan; 2 Computer System Department, Faculty of Information and Technology, Andalas University, Padang 25175, Indonesia; 3 College of Engineering, Ibaraki University, Hitachi 316-8511, Japan

**Keywords:** radiofrequency identification, sensor tags, batteryless, reduced energy consumption

## Abstract

This paper presents a feasibility study on monitoring earthquake-caused furniture vibrations using radiofrequency identification (RFID) sensor tags. Finding unstable objects by exploiting the vibrations caused by weaker earthquakes is effective as one of the potential countermeasures for large-scale earthquakes in earthquake-prone areas. For this purpose, a previously proposed ultrahigh-frequency (UHF)-band RFID-based batteryless vibration/physical shock sensing system enabled long-term monitoring. This RFID sensor system introduced standby and active modes for long-term monitoring. This system enabled lower-cost wireless vibration measurements without affecting the vibration of furniture because the RFID-based sensor tags provide lightweight, low-cost, and battery-free operations. This RFID sensor system observed earthquake-cased furniture vibrations in a room on the fourth floor of a building eight stories high at Ibaraki University, Hitachi, Ibaraki, Japan. The observation results revealed that the RFID sensor tags identified the vibrations of furniture caused by earthquakes. The RFID sensor system also observed the vibration duration times of the objects in a room and specified the most unstable reference object. Hence, the proposed vibration sensing system helped achieve safe living in indoor environments.

## 1. Introduction

Natural disasters are increasing in many parts of the world and are major obstacles to sustainable development. Reducing disasters and mitigating damages are important issues for international communities. Among the top ten natural disasters worldwide according to the death toll in 2021, earthquakes are listed as number one [1]. Japan is in the active belt of the Pacific Rim with frequent seismic activity. Compared with 0.25% of the world’s small land areas, Japan has the highest proportion of earthquakes in the world [2]. At present, many researchers have been using sensors and machine learning to prevent disasters due to earthquakes and warn people about earthquakes before they occur [3,4,5,6]. In earthquake-prone areas, the structural health monitoring (SHM) [7] of civil engineering infrastructures [8,9] is also critical. SHM introduced various sensor techniques [7]: infrared, strain gauges, relative humidity, accelerometers, global positioning systems (GPS), displacement, gas sensors, optical fibers, image sensors, pressure, ultrasonic, and moisture. In addition, machine learning techniques analyze the collected data obtained by these sensor technologies. Recently, the sensing technologies for SHM have been transitioning from wired to wireless sensing [7].

The Internet of things (IoT) has attracted much attention because it can connect things to the network using wireless IoT devices [7]. IoT devices have spread all over the world in diverse forms and applications. Especially for achieving safety and security for smart homes, IoT devices can do many things, including earthquake-caused disaster prevention [10,11,12,13]. However, due to issues with the battery life and the manufacturing costs of IoT devices, they need to overcome a series of challenges to respond to these new requirements [14,15,16,17,18,19]. Ultrahigh-frequency (UHF)-band radiofrequency identification (RFID) provides thin and small form factors, lower costs, and batteryless wireless capabilities. Hence, UHF-band RFID solves the problems of IoT devices. A basic RFID system consists of an RFID reader, an RFID tag antenna, and an RFID integrated circuit (IC) chip. The RFID reader radiates radio waves toward the RFID tags, and the RFID tags subsequently return unique electronic product codes (EPCs) to the RFID reader. RFID sensor tags have recently emerged, and they also measure the temperatures [20,21,22], humidity [23], human activity [24,25], vibrations [26], physical shocks [27], and pressures of the surrounding environments [28]. With these sensing capabilities, RFID systems can function as wireless IoT devices [29,30,31,32,33]. Inertial measurement units (IMUs) [34] and strain gauges [35] also make it possible to monitor earthquakes. However, IMUs generally use analog-to-digital converters and internal processors in addition to acceleration and gyro sensors. Strain gauges have relatively high input resistances compared with the short-circuited vibration-sensitive switches used in RFID-based vibration sensor tags. These characteristics of IMUs and strain gauges make RFID sensor tag designs difficult. A camera and ArUco markers also make it possible to measure the vibration of furniture [36,37]. However, the vibrations of a camera during earthquakes may affect the vibration-monitoring results. In contrast, the RFID reader is insensitive to vibrations and detect tiny vibrations.

Ordinary RFID sensor systems continuously transmit electromagnetic waves from an RFID reader to obtain time series of sensing data. Because earthquakes are probabilistic events that are difficult to predict, RFID sensor systems work for a long time to monitor vibrations caused by earthquakes. However, these long-term measurements lead to energy waste and temperature increases in the RFID reader. Moreover, RFID sensor systems cause considerable useless data in long-term measurements. Uploading all the resulting data to an IoT cloud is likely to be a heavy load on the network. As a result, Song et al. [38] proposed an RFID-based vibration/physical-shock sensor system for long-term measurements. This method introduced two operating modes to overcome the problems occurring in long-term measurements. These two modes were standby and active modes corresponding to low- and high-duty cycles, respectively. These two modes achieved accurate sensing without compromising the above-mentioned problems.

This paper presents a feasibility study on monitoring earthquake-caused furniture vibrations using RFID-based vibration/physical-shock sensor tags [38]. Finding unstable objects by exploiting the vibrations caused by weaker earthquakes is effective as one of the potential countermeasures for large-scale earthquakes in earthquake-prone areas. This system achieves the daily diagnostics of indoor environments. Although previous works monitored the vibrations of indoor objects and furniture [39,40,41], the proposed system enables lower-cost wireless vibration measurements without affecting the furniture vibrations because the RFID-based sensor tags provide lightweight, low-cost, and battery-free operations. The proposed earthquake-caused vibration sensing system helps achieve safe living in a home. This system enables us to find unstable objects in a house by monitoring the furniture vibration.

## 2. RFID-Based Vibration/Physical-Shock Sensor System for Long-Term Measurements

This section provides application examples for the long-term monitoring of the earthquake-caused vibration of furniture in a home. Moreover, this section explains the operational principle of an RFID sensor tag with tilt switches and the entire system structure [38].

**Figure 1:**
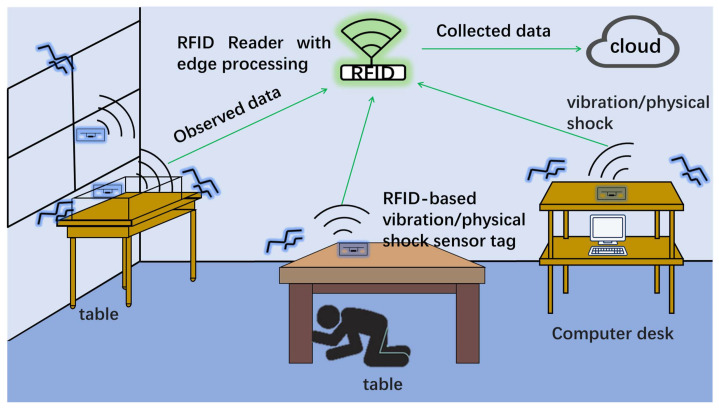
Application example for an earthquake-caused vibration monitoring system in a home.

Figure 1 shows an application example for the earthquake-caused vibration monitoring system. The vibration monitoring system monitors the earthquake-caused vibrations of a table, computer desk, and window. Table 1 compares the various environment monitoring systems with this work, summarizing the related methods mentioned in the introduction. The RFID-based vibration/physical-shock sensor system uses edge processing to enable long-term measurements. The RFID reader collects monitoring data and then uploads them to an IoT cloud. The earthquake-caused vibration sensing system allows safely living in a home because it enables us to find unstable objects.

### 2.1. RFID-Based Vibration/Physical-Shock Sensor Tag

**Figure 2:**
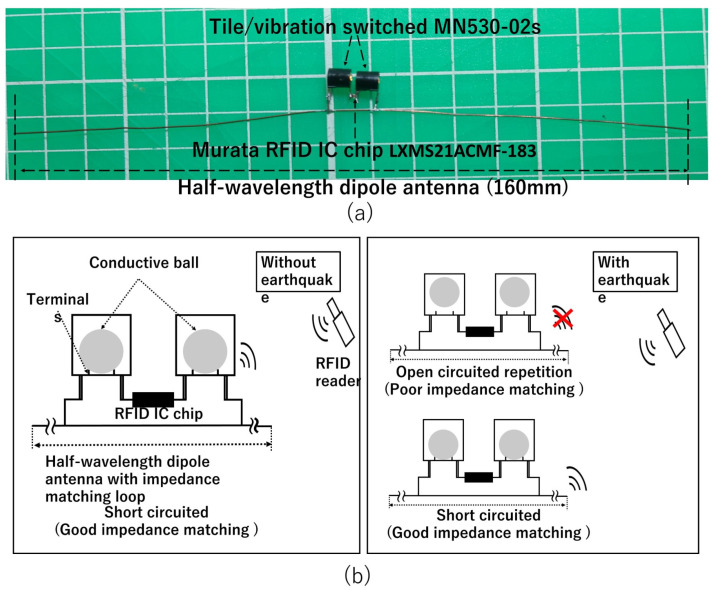
Operational principle of RFID-based vibration and physical-shock monitoring using tilt switches. (**a**) Entire RFID sensor tag; (**b**) operational principle of an RFID sensor system.

This subsection introduces an RFID-based vibration/physical-shock sensor tag. Figure 2a shows the entire RFID sensor tag, and (b) illustrates its expanded version around the feed point. The RFID sensor tag consisted of two tilt switches (G-DEVICE MN530-02S [42]), a half-wavelength dipole antenna, and an RFID IC (Murata LXMS21ACMF-183 [43]). The left and right insets in Figure 2b also illustrate the statuses of the tilt switches for the cases with and without vibrations, respectively. The tilt switch was a normally closed three-axis mechanical vibration sensor. This tilt switch caused chattering signals for applied vibrations and was sensitive to vibrations along all three axes directions. The vibrations of the internal metal boll were more than 5 Hz and detected an acceleration of more than 30 Gal [42]. An RFID reader inventoried an RFID sensor tag and counted the number of reads during the inventory. The internal metallic bolls repeatedly caused short- and open-circuited states due to vibration. These alternating statuses reduced the number of reads because the RFID sensor tag in a short-circuited state does not respond to the RFID reader due to its poor impedance-matching condition. The number of reads, therefore, became a metric for evaluating the intensities of vibrations.

### 2.2. Measurement Principle for Long-Term Monitoring

**Figure 3:**
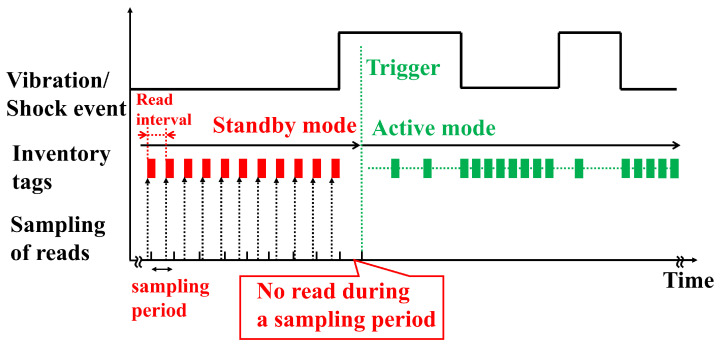
Monitoring principle for long-term measurement.

Because earthquakes are unpredictable and burst events, long-term monitoring is necessary. An event-driven sensing capability, referred to as edge processing, is demanded [38]. Figure 3 shows an operational principle of edge processing. The RFID reader has two different working states: a standby mode and an active mode.

The standby mode has a longer reading interval by employing a low-duty cycle setting [44], thereby reducing the energy consumption and operating temperature of the RFID reader [38]. That paper described employing a radio irradiation time of 50 ms in the standby mode. During ordinary times, the RFID reader worked in standby mode. The RFID reader in the standby mode sampled the RFID sensor tag readings in a relatively long sampling period, confirming that no earthquakes occurred. The sampling period was 500 ms in this paper. If the RFID reader identified the RFID sensor tag readings within a sampling period, the RFID reader continued to stay in standby mode.

The RFID reader triggered the active mode when it sampled no readings. An earthquake, therefore, altered the operating modes from standby to active. The active mode had a radio irradiation time of 200 ms by employing a high-duty cycle. A high-duty cycle increased the number of RFID sensor tag readings, enabling accurate vibration and physical-shock monitoring. By considering the characteristics of the earthquake, this paper employed an active mode duration time of 60 s.

## 3. Experiment

This section presents observation results for four earthquakes observed at the laboratory of Ibaraki University, Hitachi, Japan. Ibaraki Prefecture is one of the most earthquake-prone cities on the main island of Japan and has an average of approximately 200 earthquakes each year.

### 3.1. Software and Experiment Environment

**Figure 4:**
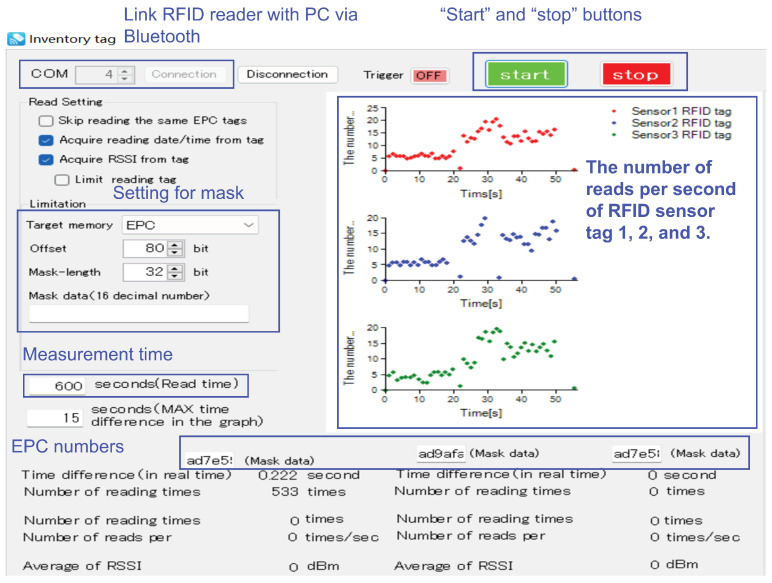
Developed RFID reader software implementing the proposed edge processing.

The measurement principle for long-term monitoring explained in the previous section was implemented in RFID reader control software. This control software program was based on a software development kit (SDK) implemented in C#. An RFID reader, DOTR-3200 [45], identified the RFID sensor tags. Figure 4 shows a user interface of the developed software application. This software program displayed observed time series metrics in real time, where the metric was the number of reads per second, and this metric was uniquely and inversely related to the intensity of vibrations and physical shocks. This control software application only kept the data for the active mode in the text files. It identified individual RFID sensor tags based on the EPC numbers.

**Figure 5:**
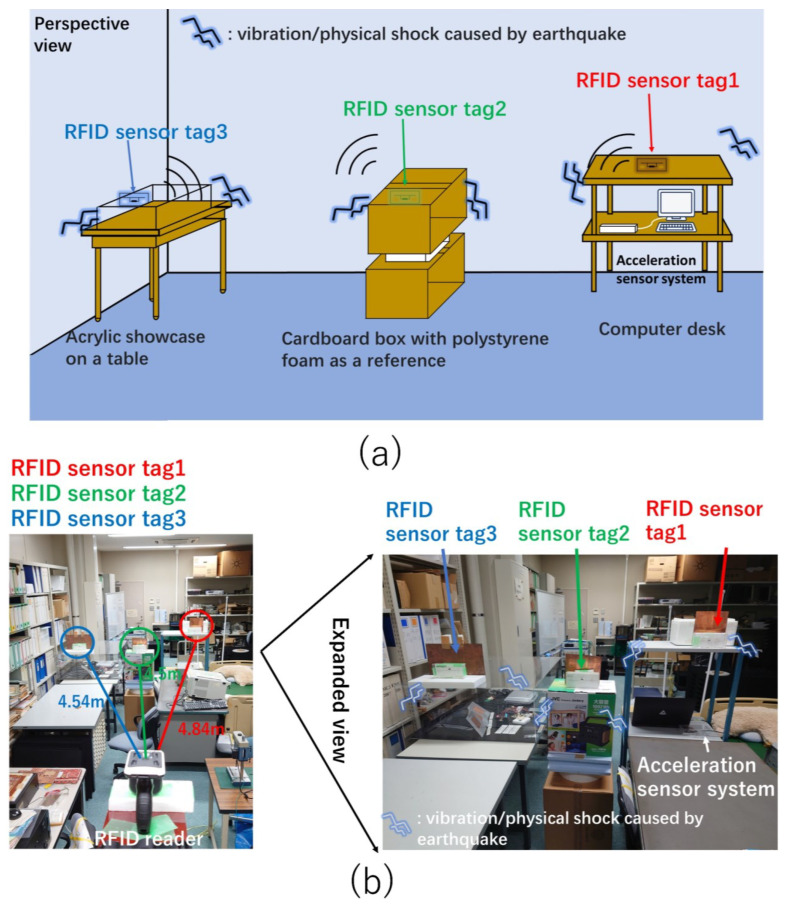
Experimental environment. (**a**) The perspective view of the three RFID sensor tags. (**b**) Photo of the experimental environment.

**Figure 6:**
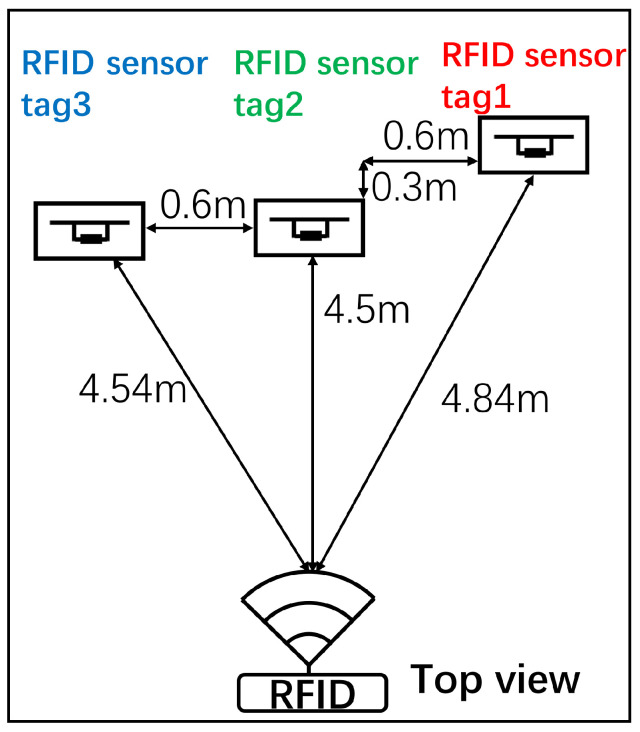
The top view and the reading distances between the RFID reader and RFID sensor tags.

RFID sensor tags were installed on furniture in a room described in this paper. An artificial object consisting of empty cardboard boxes and a rectangle made from foam polystyrene was used as an unstable reference object. Figure 5 shows the experimental setup. The three colors, red, green, and blue, differentiate RFID sensor tags. Figure 5a illustrates the experimental setup. Three objects were placed in the room: from the right, a computer desk, stacked cardboard boxes sandwiching the foam polystyrene rectangle, and a transparent acrylic showcase on a table. RFID sensor tags 1, 2, and 3 measured the vibrations and physical shocks. Figure 5b shows a photo of the experimental setup, and Figure 6 shows the top view providing the detailed arrangements of the objects and RFID reader. The RFID sensor tags had reflectors to enhance read distances, where the spacing between the RFID sensor tag antennas and the reflectors was a quarter of the wavelength at an operating frequency of 920 MHz. The maximum read range of the RFID sensor tag was approximately 4.5 m. The reflector also reduced the impact of surrounding objects and walls on the RFID sensor tag antenna.

**Figure 7:**
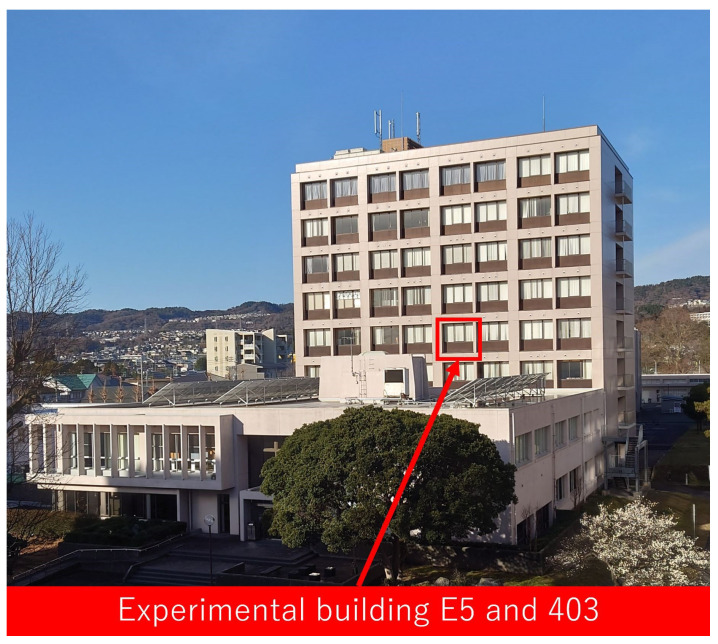
Experimental building.

The experimental room was located in the building shown in Figure 7. The building and room names were E5 and 403, respectively. The room was on the fourth floor. This building was eight stories high.

**Figure 8:**
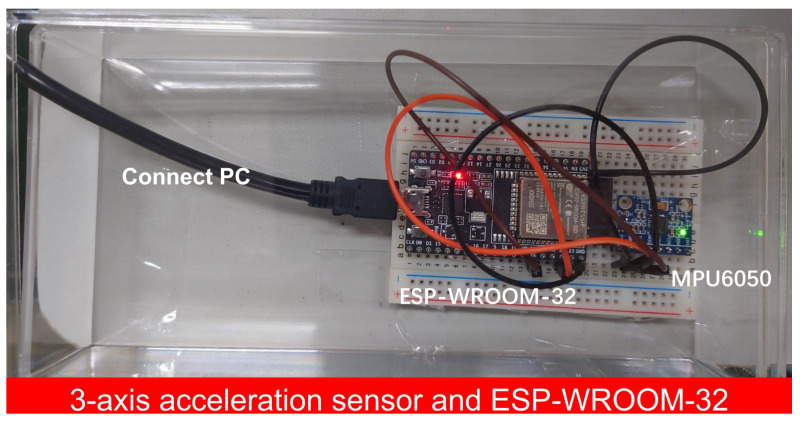
The acceleration sensor system for reference.

In this experiment, an acceleration sensor measured the vibrations and physical shocks caused by earthquakes to compare the observed data of the RFID sensor tags with those of the acceleration sensor. This acceleration sensor measured the vibration and physical shocks of the computer desk together with RFID sensor tag 1. Figure 8 shows a photo of the acceleration sensor system consisting of an acceleration sensor module, MPU6050, and microcomputer, ESP-WROOM-32. MPU6050 had digital-output three-axis accelerometers with programmable ranges of ±2 g, ±4 g, ±8 g, and ±16 g. This sensor unit also had integrated 16-bit analog-to-digital converters [46]. These features enabled accurate motion detection. This sensor unit was used for camera stabilization. These characteristics led to the minimum acceleration of 0.06 Gal. Hence, this sensor was suitable for evaluating the RFID sensor tag using the tilt switches. The root sum square of the three axes data was used to evaluate the accelerations. Windows Time service was used to adjust the time of a computer, thus achieving time synchronization between the MPU6050 and the RFID reader. Earthquake occurrence times issued by the Japan Meteorological Agency helped distinguish the vibrations of earthquakes from unexpected human activities.

### 3.2. Observation Results

**Figure 9:**
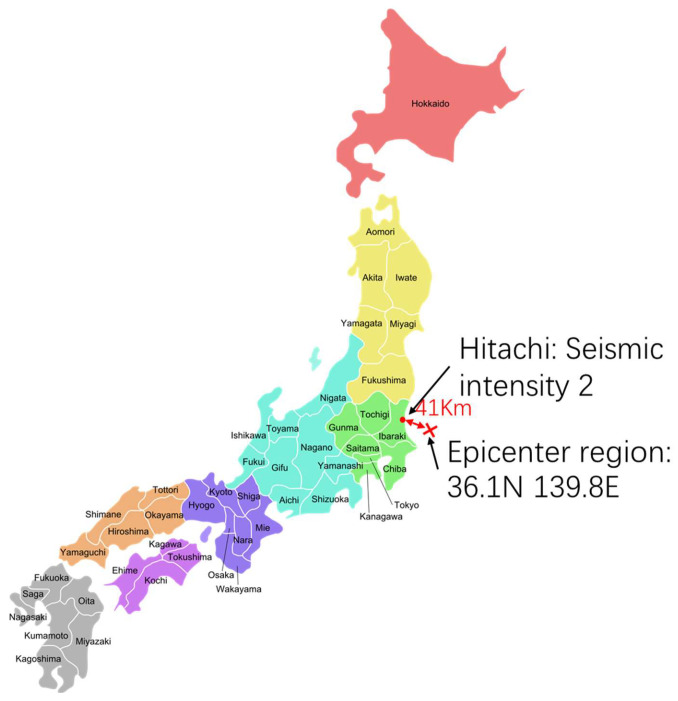
The maximum seismic intensity in Hitachi and the distance between Hitachi and the epicenter region off the coast of Ibaraki Prefecture.

Figure 9 shows the observed results for the earthquake on 7 December 2022, at 5:40 JST. The epicenter region was off the coast of Ibaraki Prefecture, and the epicenter was 41 km away from Hitachi. The seismic magnitude scale was four. The seismic intensity in Hitachi was two.

**Figure 10:**
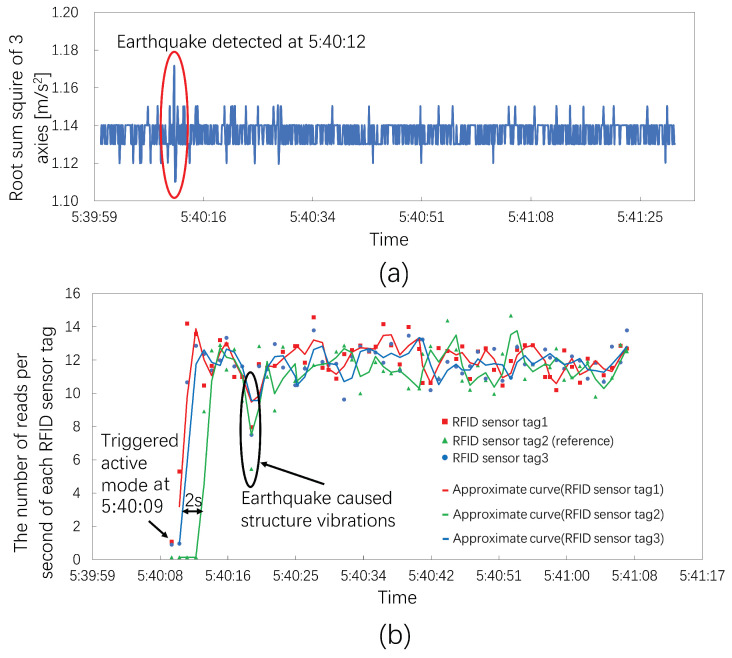
The number of reads per second for three RFID sensor tags at an earthquake seismic intensity of 2. (**a**) Root sum squares of the acceleration sensor system; the acceleration sensor system detected the earthquake at 5:40:12. (**b**) The number of reads per second of each RFID sensor tag; the RFID sensor tags triggered the active mode at 15:40:09.

The acceleration sensor system observed a vibration at 5:40:12 JST, as shown in Figure 10a. Figure 10b shows the results of the three RIFD sensor tags. The RFID sensor system triggered the active mode at 5:40:09 JST.

The RFID sensor tags had higher detection accuracies because they triggered the active mode three seconds before being detected by the acceleration sensor. RFID sensor tags 1 and 3 returned to ordinary levels at a similar time. RFID sensor tag 2 also returned to the ordinary level but 2 s after RFID sensor tags 1 and 3 because RFID sensor tag 2 monitored the unstable reference object. In addition, all the RFID sensor tags also detected vibrations at the time indicated by a black elliptical circle during the active mode. At this time, RFID sensor tags 1 and 3 had comparable metric values, and RFID sensor tag 2 detected a much lower value. These natural results confirmed that the reference object was the most unstable, and the computer desk and table with the acrylic showcase had comparable vibrations.

**Figure 11:**
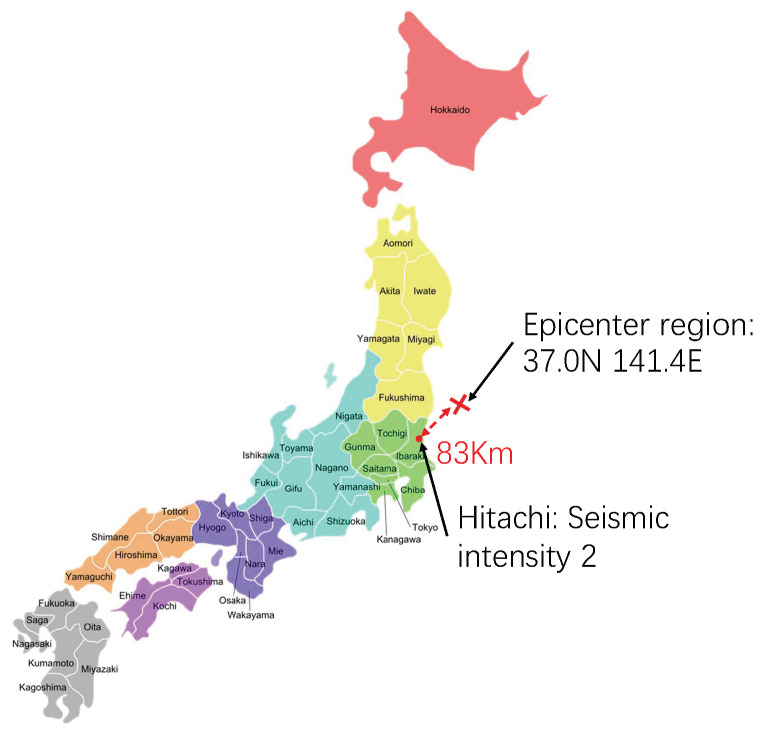
The maximum seismic intensity in Hitachi and the distance between Hitachi and the epicenter region off the coast of Fukushima Prefecture.

**Figure 12:**
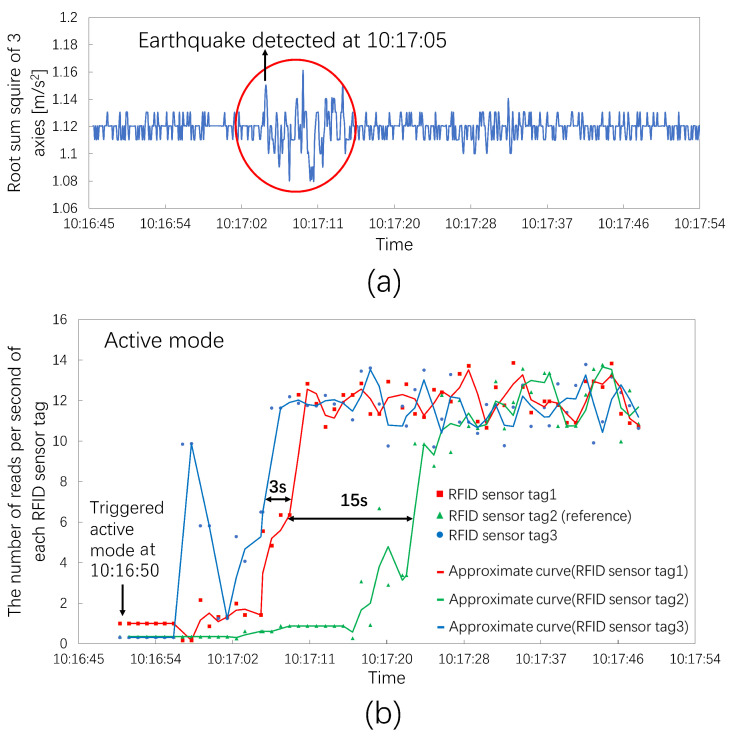
The number of reads per second of three RFID sensor tags at an earthquake seismic intensity of two. (**a**) The root sum square of the acceleration sensor system; the acceleration sensor system detected the earthquake at 10:17:05. (**b**) The number of reads per second of each RFID sensor tag in this experiment; the RFID sensor tags triggered the active mode at 10:16:50.

Figure 11 shows the observed results for the earthquake on 8 January 2023, at 10:16 JST. The epicenter region was off the coast of Fukushima Prefecture, and the epicenter was 83 km away from Hitachi. The seismic magnitude scale was 4.7. The seismic intensity in Hitachi was two. The acceleration sensor system observed vibration at 10:17:05 JST, as shown in Figure 12a. Figure 12b shows the results for the three RIFD sensor tags. The RFID sensor system triggered the active mode at 10:16:50 JST, just 15 s earlier than the acceleration sensor. The metric values of RFID sensor tag 3 first returned to the ordinary level, and those of RFID sensor tag 1 subsequently followed RFID sensor tag 3 approximately 3 s later. The metric values of RFID sensor tag 2 monitoring the reference object finally increased to the ordinary level 15 s later.

**Figure 13:**
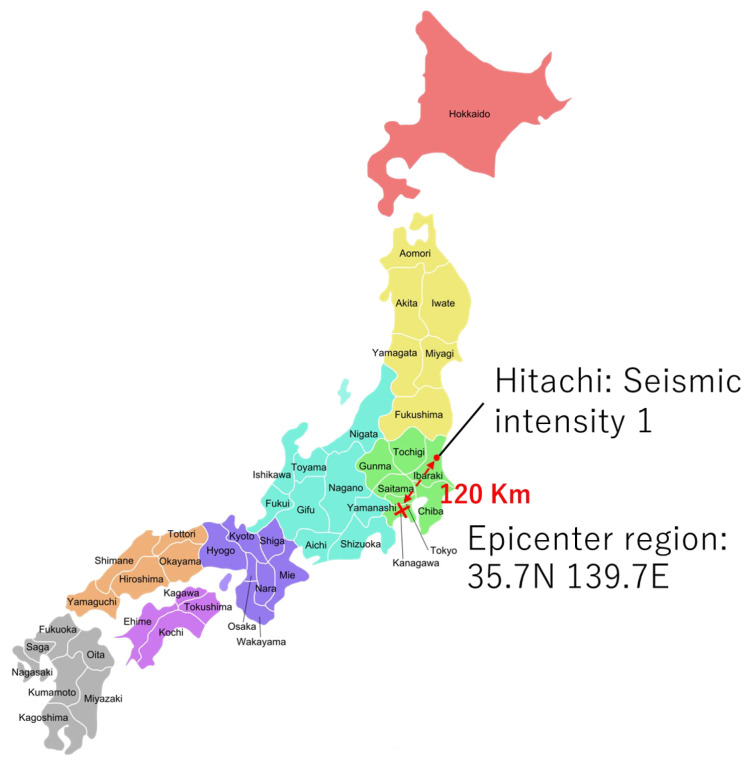
The maximum seismic intensity in Hitachi and the distance between Hitachi and the epicenter region, 23 wards, Tokyo.

Figure 13 shows the observed results for the earthquake on 10 December 2022, at 6:37 JST. The epicenter region was in 23 wards, Tokyo, and the epicenter was 120 km away from Hitachi. The seismic magnitude scale was four. The seismic intensity in Hitachi was one.

**Figure 14:**
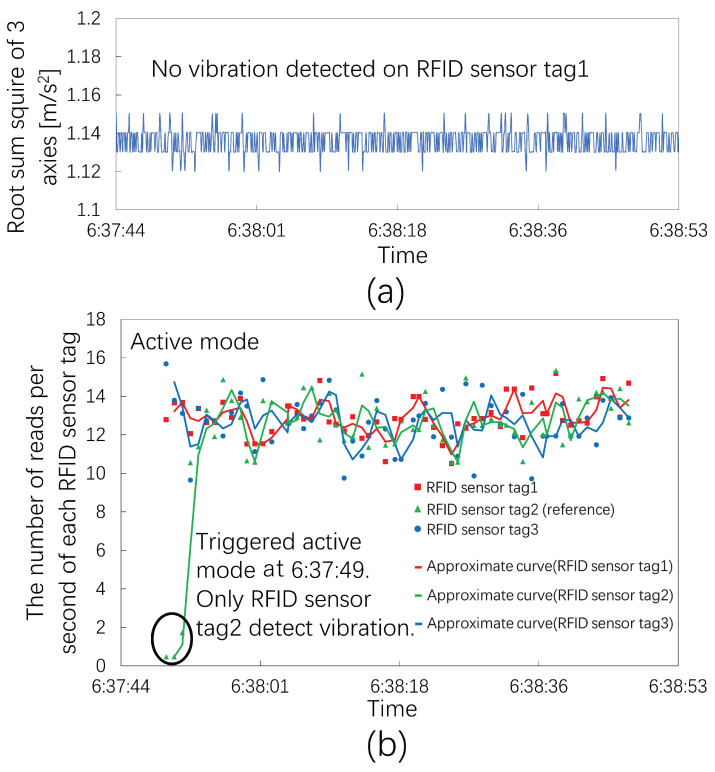
The number of reads per second of three RFID sensor tags at an earthquake seismic intensity of 1. (**a**) The root sum square of the accelerated sensor system and no vibration detected on RFID sensor tag 1. (**b**) The number of reads per second of each RFID sensor tag in that experiment.

The acceleration sensor system did not observe any vibrations, as shown in Figure 14a. Figure 14b shows the results for the three RIFD sensor tags. RFID sensor tag 2 triggered the active mode at 6:37:49 JST.

The acceleration sensor system and RFID sensor tags 1 and 3 did not detect vibrations because the seismic intensity was weak. Only RFID sensor tag 2 on the reference object detected vibrations. Therefore, the unstable reference object enabled us to confirm the sensitivity of the RFID sensor tag because a seismic intensity of one was the minimum indicator. Comparing experimental results in Figure 10 and Figure 12 with Figure 14 validated that the RFID sensor tags detected the vibration of furniture for a seismic intensity of two or more.

**Figure 15:**
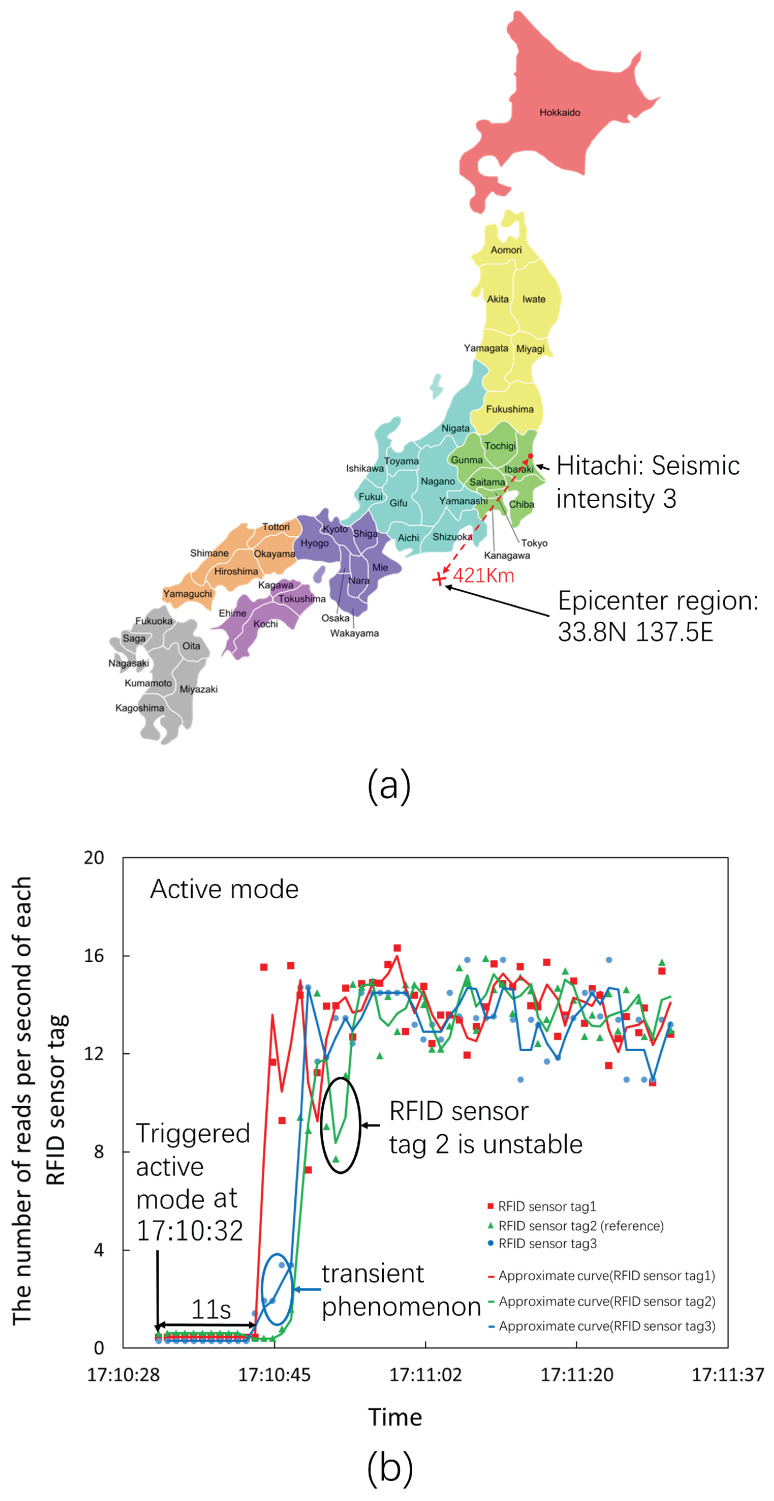
The number of reads per second of three RFID sensor tags at an earthquake seismic intensity of three. (**a**) The epicenter region and the seismic intensity in Hitachi; (**b**) the number of reads per second of each RFID sensor tag; the RFID sensor tags triggered the active mode at 17:10:32.

Figure 15a shows the observed results for the earthquake on 14 November 2022, at 17:09 JST. The epicenter region was on the southeast coast of Mie Prefecture, and the epicenter was 421 km away from Hitachi. The seismic magnitude scale was 6.1. The seismic intensity in Hitachi was three.

Figure 15b shows the time series data obtained by the three RFID sensor tags. These RFID sensor tags triggered the active mode at 17:10:32. The metric of RFID sensor tag 1 first returned to the ordinary level 11 s after the beginning of the active mode. The metric of RFID sensor tag 3 started to rise at almost the same time as that of RFID sensor tag 1 and then returned to the ordinary level through an obvious transient response. Finally, the metric of the reference RFID sensor tag 2 returned to the ordinary level through an unstable transient response. These results also confirmed that the object monitored by RFID sensor tag 2 was the most unstable in that observation.

## 4. Conclusions

This paper presented a feasibility study on monitoring earthquake-caused furniture vibrations using RFID sensor tags. A batteryless earthquake vibration sensing system based on UHF-band RFID observed the vibration of furniture during earthquakes. Observations in this paper exhibited the employment of the standby and active modes for monitoring vibrations for a long time. These modes achieved accurate vibration and physical-shock sensing while reducing the temperature rise, energy consumption, and quantity of data of an RFID reader [38]. Finding unstable objects in indoor environments by exploiting weaker earthquakes is effective as one of the potential countermeasures for large-scale earthquakes in earthquake-prone areas. The proposed system enabled lower-cost wireless vibration measurements without affecting the furniture vibrations because the RFID-based sensor tags provided lightweight, low-cost, and battery-free operations. By observing actual earthquakes, the RFID sensor system enabling long-term measurements could monitor the earthquake-caused furniture vibrations. Several observations also confirmed that the RFID sensor system with sensitive tilt/vibration switches provided a more sensitive sensing capability than a commercially available acceleration sensor. The RFID sensor system enabled the identification of unstable furniture. The RFID sensor system observed the vibration duration times of furniture.

The observed results also validated that the RFID sensor system monitored the vibration of furniture for a seismic intensity of two or more. The observed results verified that higher seismic intensities led to lower metrics and longer vibration duration times. This finding is crucial for building monitoring systems and achieving safe living in indoor environments. RFID sensor tags are easy to install on furniture because they have no internal battery. Therefore, monitoring the vibrations of furniture makes it possible to achieve safe living in a home.

## Figures and Tables

**Table 1:** Comparison of the environmental observation systems.

Method	Principles and Objectives
SHM [7]	This system monitors adverse structural changes, achieving reliability and life cycle management. This has been widely used in various civil engineering fields.
IoT [10,11,12,13]	IoT technologies collect data on vibrations, physical shocks, temperatures, and humidity and upload the data to IoT clouds.
IMU [34]	This system combines an accelerometer and gyro sensor, monitoring the accelerations and rotations of motions. IMU monitors building structures.
Strain gauges [35]	This system measures the strain force on the beam to evaluate the seismic performance of the building.
ArUco markers [36,37]	These systems use industrial cameras to measure the displacements of building structures, analyzing their vibrations.
RFID [20,21,22,23,24,25,26,27,28]	They combine various sensors with RFID systems to realize low-cost and batteryless wireless sensing.
This work	An RFID-based batteryless vibration/physical-shock sensing system for long-term monitoring [38] enables the detection of earthquake-caused furniture vibrations. Finding unstable objects by exploiting the vibrations caused by weaker earthquakes is effective as one of the potential countermeasures for large-scale earthquakes in earthquake-prone areas.

## Data Availability

Not applicable.
